# Identification and specificity of broadly neutralizing antibodies against HIV

**DOI:** 10.1111/imr.12484

**Published:** 2017-01-30

**Authors:** Laura E. McCoy, Dennis R. Burton

**Affiliations:** ^1^ Department of Immunology & Microbial ScienceIAVI Neutralizing Antibody CenterCenter for HIV/AIDS Vaccine Immunology and Immunogen DiscoveryThe Scripps Research InstituteLa JollaCAUSA; ^2^ Division of Infection & ImmunityUniversity College LondonLondonUK; ^3^ Ragon Institute of Massachusetts General HospitalMassachusetts Institute of Technology and Harvard UniversityCambridgeMAUSA

**Keywords:** B cells, HIV, monoclonal antibody isolation, neutralization

## Abstract

Beginning in 2009, studies of the humoral responses of HIV‐positive individuals have led to the identification of scores, if not hundreds, of antibodies that are both broadly reactive and potently neutralizing. This development has provided renewed impetus toward an HIV vaccine and led directly to the development of novel immunogens. Advances in identification of donors with the most potent and broad anti‐HIV serum neutralizing responses were crucial in this effort. Equally, development of methods for the rapid generation of human antibodies from these donors was pivotal. Primarily these methods comprise single B‐cell culture coupled to high‐throughput neutralization screening and flow cytometry‐based sorting of single B cells using HIV envelope protein baits. In this review, the advantages and disadvantages of these methodologies are discussed in the context of the specificities targeted by individual antibodies and the need for further improvements to evaluate HIV vaccine candidates.

This article is part of a series of reviews covering B cells and Immunity to HIV appearing in Volume 275 of *Immunological Reviews*.

## 1 Introduction

Natural immunity to many viral diseases relies upon circulating neutralizing antibodies from long‐lived plasma cells in the bone marrow or the production of neutralizing antibodies from memory B cells after re‐activation by the infecting pathogen, frequently years after the original exposure. Successful vaccines such as that for smallpox present a non‐pathogenic form of the infectious agent and induce a similar natural immunity. For HIV, however, natural immunity appears ineffective. Thus, for example, superinfection occurs unhindered by HIV envelope protein (Env)‐specific antibodies,^1^ the majority of which are non‐neutralizing.^2^ However, given that the mechanism of viral protection and clearance by antibodies in vivo is so widespread, we, and others, have studied humoral responses in HIV‐infected donors for more than two decades to understand how to prevent and control HIV infection. This persistence has led to the identification of many broadly neutralizing antibodies (bnAbs),^3^, ^4^, ^5^, ^6^ which, although relatively rare in HIV‐infected individuals,^7^, ^8^ are nevertheless highly effective against most circulating strains and can prevent infection in robust animal models.^9^, ^10^, ^11^ Therefore, although HIV infection does not induce protective antibody‐mediated immunity, it is possible for the human immune system to produce antibodies that may, in principle, protect from HIV infection. This review will focus on the epitopes targeted by bnAbs and the methodologies used to identify them. In particular, as requested, we concentrate on our own efforts in the field with important developments in other laboratories included.

The first HIV bnAbs were isolated by our laboratory using phage display^12^, ^13^, ^14^ and by Hermann Katinger's laboratory using human hybridoma electrofusion.^15^, ^16^ These were the bnAbs b12 and 2F5. Later, the bnAbs 2G12 and 4E10 were described.^17^, ^18^, ^19^ However, although these bnAbs proved very useful in answering questions about the interplay of HIV and nAbs, there was a definite lull in isolating new bnAbs. High‐throughput neutralization assays were a major factor in changing that situation. The ability to analyze mAb and serum activity against large panels of viruses was demonstrated^20^ and subsequently used to evaluate large numbers of HIV‐infected donors in the International AIDS Vaccine Initiative (IAVI) Protocol G and C studies to identify those with exceptionally potent and broad sera,^8^ map the specificities underlying these responses,^7^, ^21^ and then isolate bnAbs from these individuals.^22^, ^23^, ^24^, ^25^, ^26^, ^27^, ^28^ Independently, the standardization of the TZM‐bl neutralization assay and the definition of neutralization sensitivity tiers^29^, ^30^, ^31^ allowed much more rigorous serum analysis.

**Figure 1:**
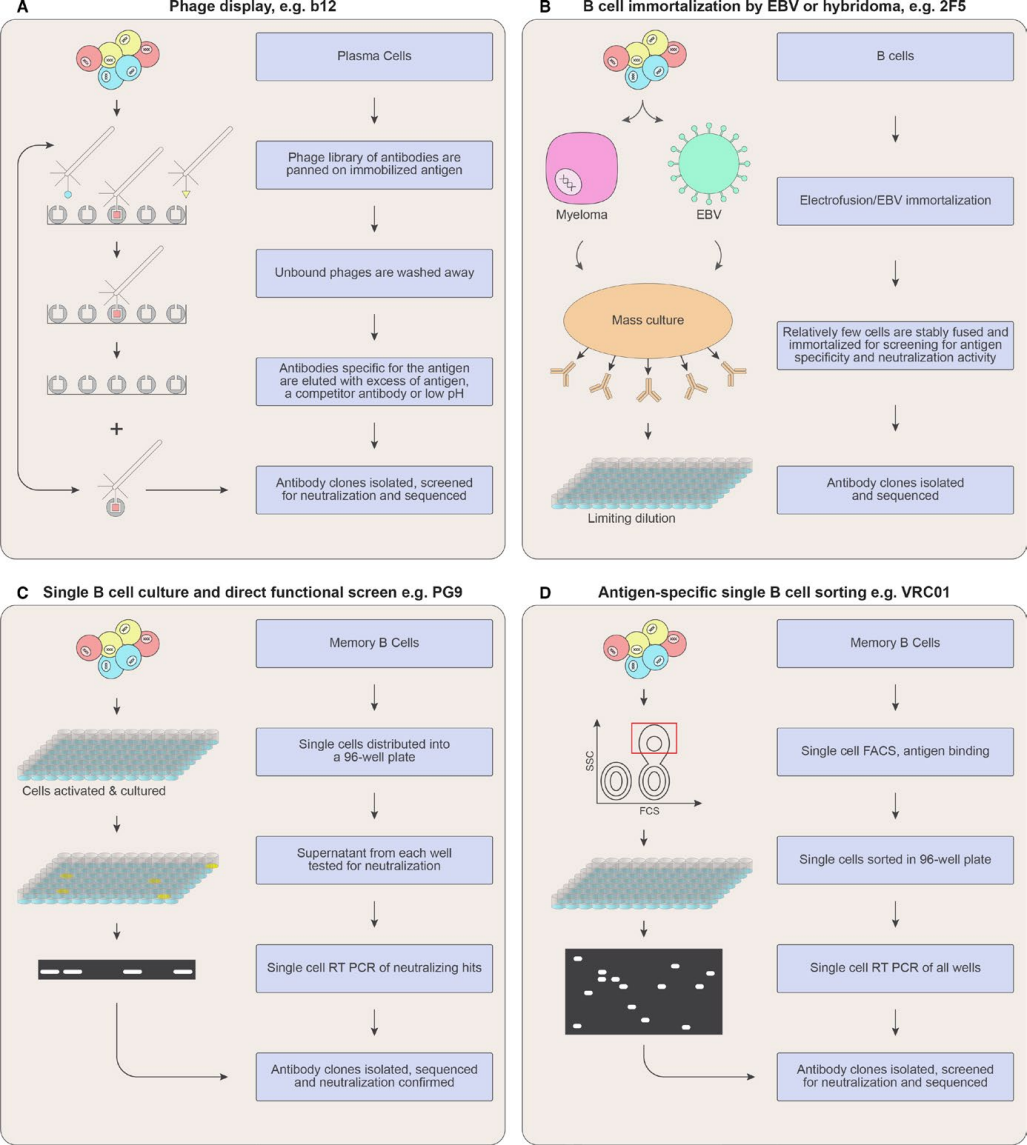
Methods for HIV bnAb isolation. (A) mAb isolation by phage library from plasma cells and subsequent phage display to enrich for antigen‐specific clones; (B) mAb isolation by immortalization of total B cells. Propagated cells are then serially diluted and Abs secreted in the supernatant tested for antigen specificity; (C) mAb isolation by single B‐cell culture without immortalization, Abs secreted in the supernatant tested for antigen specificity and Ab sequences obtained; (D) mAb isolation by antigen‐specific single B‐cell FACS. Ab sequences are amplified from each well and tested for antigen specificity

A second major factor in generating new bnAbs was the development of single B‐cell approaches for the isolation of human antibodies^32^, ^33^, ^34^ (Figure 1). Beginning with the description of bnAbs PG9 and PG16 in 2009, the field saw a revolution in the generation of bnAbs and in parallel the development of ever improving tools for the analysis of the specificities of these Abs. Structural tools, crystallography and cryo‐electron microscopy, have been critical as have biophysical and virological approaches.

## 2 Identification of HIV bnAbs

An important step in the identification of HIV bnAbs was the ability to study large cohorts and identify those with potent and broad serum neutralizing activity. This was first achieved by defining criteria to rank 1800 HIV‐positive serum samples from the IAVI Protocol G cohort for broad and potent activity against relatively neutralization‐resistant isolates to represent circulating viruses.^8^, ^21^ We selected and validated a six‐virus cross‐clade indicator panel and developed a scoring system wherein elite activity is defined as neutralization of at least one virus with an IC_50_ value of more than 1:300 across a minimum of four different clades.^8^ Having identified HIV‐positive donors with broad and potent neutralizing activity, the next step was to isolate the monoclonal Abs (mAbs) giving rise to this phenotype. Given the limited efficiency of both B‐cell immortalization and phage display, we opted to directly screen stimulated single B‐cell supernatants. This approach used a strategy that required adaption to a high‐throughput format to screen enough B cells to identify rare HIV bnAbs (Figure 1).

In the first experiment, we screened more than 30 000 individual B cells from one donor for the ability to neutralize two HIV strains and also bind to recombinant gp120 and gp41 protein subunits^23^ (Table 1). This screen yielded five B‐cell clones which produced mAbs with the ability to neutralize at least one HIV strain, where one was a neutralization‐resistant Tier 2 isolate JR‐CSF and the other a highly neutralization‐sensitive Tier 1 strain SF162. Interestingly, only two of the five mAbs, the somatic variants PG9 and PG16, potently neutralized JR‐CSF. Unlike the other three mAbs generated, PG9 and PG16 did not neutralize SF162 nor bind to the recombinant Env subunits. Thus, this validated the utility of a screening method in which the primary selection criterion is neutralization of Tier 2 strains of HIV, because a method relying first on binding activity, such as phage display or B‐cell sorting, relying on binding activity of existing antigens such as gp120, would likely have failed to identify the bnAbs PG9 and PG16 that neutralize 73%–78% of strains tested. Furthermore, that only five neutralizing mAbs were isolated from 30 000 single B‐cell cultures derived from a donor with a favorable serum neutralizing profile validated the use of high‐throughput screening to identify rare bnAbs.

**Table 1:** BnAb specificity and isolation method

BnAb	Epitope	Isolation method
PG9	Apex	B‐cell culture/neutralization
PG16	Apex	B‐cell culture/neutralization
PGT145	Apex	B‐cell culture/neutralization
PGDM1400	Apex	B‐cell selection/antigen binding
CAP256.VRC26	Apex	B‐cell culture/neutralization
CH01	Apex	B‐cell culture/neutralization
PGT121	High‐mannose patch	B‐cell culture/neutralization
PGT128	High‐mannose patch	B‐cell culture/neutralization
PGT135	High‐mannose patch	B‐cell culture/neutralization
10‐1074	High‐mannose patch	B‐cell selection/antigen binding
VRC01	CD4bs	B‐cell selection/antigen binding
CH103	CD4bs	B‐cell selection/antigen binding
3BNC117	CD4bs	B‐cell selection/antigen binding
PGV04	CD4bs	B‐cell selection/antigen binding
8ANC131	CD4bs	B‐cell selection/antigen binding
CH235	CD4bs	B‐cell culture/neutralization
PGT151	gp120‐gp41 interface	B‐cell culture/neutralization
35022	gp120‐gp41 interface	B‐cell culture/neutralization
8ANC195	gp120‐gp41 interface	B‐cell selection/antigen binding
ACS202	gp120‐gp41 interface	B‐cell selection/antigen binding
N123‐VRC34.01	gp120‐gp41 interface	B‐cell selection/antigen binding
10E8	MPER	B‐cell culture/neutralization

This approach of high‐throughput neutralization screening of single B‐cell cultures was re‐employed to isolate bnAbs from multiple donors (Table 1). These bnAbs comprise the PGT121, PGT128, PGT135, PGT145, and PGT151 families,^22^, ^25^ which are among the most potent bnAbs isolated to date, with PGT121 found to be protective at low doses in an in vivo challenge model.^35^ Similar large single B‐cell culture screens led to the identification of the highly potent and broad 10E8, a gp41 membrane proximal region (MPER)‐specific bnAb^36^ and 35022, which binds the gp120‐gp41 interface,^37^ from the same donor. All of these studies operated on the basis that the neutralization specificity of the donor serum was unknown, and therefore, any Ab isolation method should not be biased by the use of pre‐enrichment for binding activity.

However, simultaneous advances in our ability to discern the specificities that mediate elite neutralization^21^ advocated for the use of a recombinant Env protein as bait for bnAb B cells. This method was used to identify multiple Ab lineages from six donors but did not identify any with bnAb activity.^38^ In contrast, the bnAb VRC01 was identified using a resurfaced Env gp120 subunit (RSC3) bait that preferentially bound the previously isolated bnAb b12 and was recognized by the individual donor's neutralizing serum^39^ (Figure 1, Table 1). RSC3 was fluorescently labeled and mixed with donor cells so that RSC3‐positive B cells could be separated by fluorescence‐activated cell sorting (FACS) into individual wells. cDNA was generated from each well and heavy‐ and light‐chain pairs cloned, recombinantly expressed and then screened for neutralization activity. Similar approaches with different baits were used to isolated additional gp120‐specific bnAbs including 3BNC117, 3BNC60,^40^ and 10‐1074.^41^ In this method, selection is based purely on the ability to bind the Env bait, and many non‐neutralizing mAbs may also be cloned unless there is counter selection with an epitope‐specific knockout probe. We successfully used this approach to isolate the PCDN series of bnAbs from a protocol C donor, whose serum neutralization activity was N332 dependent, enabling counter selection with a 332 glycan knockout negative probe.^26^ However, it should be noted that this strategy selects for bnAbs with a stringent requirement for an N332 glycan and will not select for antibodies that can use nearby glycans interchangeably with the N332 glycan, as found for the PGT121 family bnAbs.^42^

Many of the bnAbs identified by screening B‐cell cultures are trimer‐preferring or ‐specific, such as PG9 and PGT151, and as such, they may not be identified by antigen selection using molecules such as monomeric gp120 (Table 1). It should be noted, however, that the bnAb 8ANC195 was isolated by selection with a gp120 core protein^40^ but was later revealed to bind an area spanning the gp120‐gp140 interface rather than exclusively gp120.^43^ To counter the limitation of binding selection based on the gp120 subunit, Env on the surface of cells was used as a bait to select the bnAbs 3BC315 and 3BC176,^44^ which are also specific for the gp120‐gp41 trimer interface.^45^ Subsequently, advances in the production of soluble near‐native stabilized Env trimers^46^ have allowed better selection of Env‐specific B cells, excluding those which bind regions not exposed on the infectious viral spike. Using this method, we isolated PGDM1400^28^ from the same donor that previously yielded the PGT141–145 family of bnAbs via the B‐cell culturing approach.^22^ Furthermore, stabilized BG505 SOSIP.664 trimers have been used as baits to isolate two bnAbs which occupy overlapping epitopes at the gp120‐gp41 interface and also contact the fusion peptide, ACS202^47^ and VRC34.^48^ Similarly, additional apex‐specific bnAbs were recently isolated from the CAP256 donor using both the BG505 SOSIP.664 trimer and B‐cell culture.^49^ However, the most potent new bnAb was found by the latter method, leading the authors to emphasize the advantages of this method.^49^

Importantly, the isolation and characterization of bnAbs have occurred concurrently with B‐cell ontogeny studies that have, in turn, suggested novel ways to identify additional bnAbs. Next‐generation sequencing (NGS) data generated from total RNA from PGT121/4 donor lymphocytes revealed an extensive family tree of possible PGT121/4 heavy‐ and light‐chain combinations with as little as 6% amino acid mutation but still notable neutralization breadth.^50^ Studies by other groups have identified bnAbs including CAP256,^51^ CH103,^52^ and CH235^53^ that highlight the extensive viral epitope diversification and interplay between B‐cell lineages during the co‐evolution of virus and bnAbs. Similarly, our study of an N332‐dependent Protocol C donor yielded a family of bnAbs and precursors from 16 to 38 months post infection.^26^ The development of the bespoke Ab analysis platform Clonify^54^ enabled us to filter these data for PCDN Abs and identify a likely unmutated common ancestor (UCA) of the lineage and revealed that there was a virus‐triggered selection bottleneck in Ab maturation after 27 months. Thus, the application of NGS techniques to study B‐cell repertoires from peripheral lymphocytes has greatly increased our understanding of bnAb development. However, it is important to note that at least one individual bnAb must first be identified and validated experimentally; otherwise, it is generally not possible to decipher which rare B‐cell transcripts encode bnAbs. There have been attempts to mine NGS B‐cell repertoire data by predicting heavy‐ and light‐chain pairing^55^ but paired heavy and light sequencing technology will be required to gain a clearer understanding of bnAb donor repertoires. Furthermore, VRC01‐class bnAbs have been found in multiple donors and carry certain genetic hallmarks. However, given there can be up to 50% sequence divergence between Abs from different individuals,^56^, ^57^ it is challenging for NGS alone to identify new bnAbs even within this class.^58^ Experimentally, this meant pre‐screening of VRC01‐like heavy chains paired with the original light chain of VRC01 was required. Heavy chains, which were functional, were then paired with donor light chains encoding the characteristic five‐amino‐acid sequence motif of VRC01.^58^ However, such an approach is not trivial and is not applicable to donors with undefined specificities.

## 3 Specificity of HIV bnAbs

The ability to identify bnAbs over the last decade has dramatically increased our knowledge of the specificities underlying broad and potent neutralization of HIV.^3^ In turn, this has allowed more thorough pre‐screening of potential bnAb donor serum samples. If the specificity of a neutralizing response can be determined, it can help to decide whether a binding or neutralization‐based selection method is the best option to isolate bnAbs (Table 1). There is also now a greater understanding of the frequency and distribution of bnAb epitopes, both within individual patients and across cohorts.^59^ However, there may remain additional epitopes to identify as shown by the serological analysis of protocol C, the most diverse longitudinal primary infection cohort studied to date. This study revealed that the bnAb specificity of 12% of the 439 donors is unknown.^7^ Where the specificity could be determined in the top‐ranking neutralizing donors, the majority of bnAb specificities mapped to glycan‐dependent epitopes, including the apex, high‐mannose patch, and PGT151‐like gp120‐gp41 interface epitopes.^7^ It is especially noteworthy that all of these epitopes were originally defined by bnAbs discovered by direct neutralization screening rather than antigen selection (Table 1). Only one top‐ranking neutralizing donor exhibited a CD4‐binding site dependent bnAb response, while many donors made type‐specific CD4‐binding site responses,^7^ in agreement with other studies.^60^ However, it should be noted that CD4‐binding site bnAbs typically display very high levels of somatic hypermutation, which may necessitate a longer post‐infection time period to develop than typically studied. Indeed, CD4‐binding site bnAb activity emerged in only one subject at 66 months in the protocol C study.^7^ Of note, other studies have suggested a greater proportion of bnAb serum responses to target the CD4‐binding site.^21^, ^61^, ^62^

Thus, while the serum neutralizing specificity of new donors can often be identified, there may be cases where serum profiles appear similar to previously studied donors, but the nuances of a particular individual's bnAb response differ. In turn, this may mean that a typical isolation strategy could risk missing novel bnAbs that target known epitopes in different ways. This idea is suggested by the complexity with which existing bnAb families target their shared epitopes in subtly different ways. These differences are outlined below for the major classes identified to date, namely, those targeting the trimer apex, high‐mannose patch, CD4‐binding site, gp120‐gp41 interface, and MPER.

**Figure 2:**
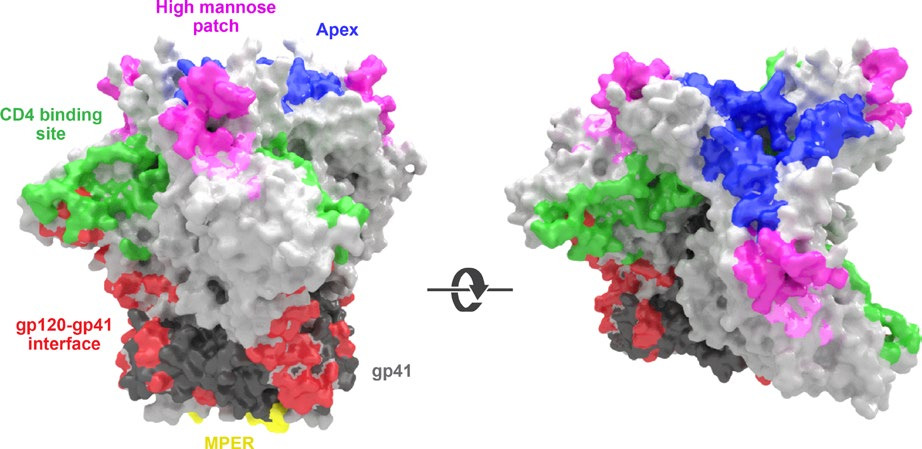
Epitope regions targeted by HIV bnAbs. Model based on the fully glycosylated BG505 SOSIP.664 trimer constructed using PDB: 4ZMJ.^103^ The gp120 and gp41 subunits are colored light gray and dark grey respectively. The five bnAb epitope regions are labeled as follows: the apex site is colored purple, the high‐mannose patch is colored magenta, the CD4bs is colored green, the gp120‐gp41 region is colored red, and MPER is colored yellow

The first class of bnAbs targeting a shared site, but with subtle differences in the exact epitope bound, is the trimer apex‐binding Abs (Figure 2). The pioneering examples of this class are PG9 and PG16,^23^ which we showed bind to a novel trimer‐preferring, glycan‐dependent bnAb epitope.^63^, ^64^, ^65^ The glycan site at residue 160 is typically critical for these bnAbs and a decrease in neutralization is seen when additional glycan sites are removed from the V1, V2, and V3 loops in a viral isolate‐dependent manner.^66^ With the isolation of additional N160‐dependent apex bnAbs, by our group and others,^22^, ^42^, ^51^, ^67^ this class can be divided into four groups typified by the prototypes PG9, CH01, PGT145, and CAP256.VRC26.09 (CAP256.09).^68^ All four prototypes bind N160 and basic residues in the lysine‐rich strand C of the V2 loop, but the exact residues required for each epitope vary, with a lysine at position 169 the most commonly shared feature.^68^ Furthermore, while N160 is absolutely required for only three out of four prototypes, CAP256.09 is only partially dependent on a glycan at this position.^51^, ^68^ There are also differences in the particular glycans preferred by each prototype, with variations even between PG9 and PG16, which prefer glycans with α‐2‐3 and α‐2‐6 linked sialic acid terminal sugars, respectively.^68^, ^69^ In addition, we found that virus produced in the presence of kifunensine, resulting in untrimmed high‐mannose glycans, is not neutralized by PG9/16.^66^ These bnAbs are also sensitive to natural glycan heterogeneity, which means a fraction of virions may be resistant to neutralization because they contain glycoforms that are not recognized by the Abs, resulting in incomplete neutralization curves.^66^ We have also observed this phenomenon with additional apex bnAbs such as the potent PGDM1400^28^ and also many other bnAb specificities. This effect varies with different bnAb and viral isolate combinations.^70^ However, the extent to which incomplete neutralization is observed with serum samples remains to be determined.

The second class of bnAbs to consider is made up of those binding the high‐mannose patch on the gp120 subunit of the Env trimer (Figure 2).^22^, ^71^ As per the apex bnAbs, we identified this class by screening single B‐cell cultures, which led to the isolation of the PGT121/4, PGT128, and PGT135 families from three individual donors.^22^ Later epitope‐focused binding‐based screens yielded similar bnAbs.^26^, ^27^, ^41^ These bnAbs were shown to compete with 2G12, to lose binding activity upon EndoH deglycosylation^22^ and to bind to the N332 glycan and a gp120 protein epitope including the sequence GDIR.^22^, ^27^, ^72^ By comparing structural information generated for different families within this class, it was shown that the N332/GDIR epitope is accessed from a variety of angles by the different bnAbs, which use diverse binding modes, leading to its definition as a supersite of vulnerability.^71^ Furthermore, in contrast to some other bnAb classes, these high‐mannose patch bnAbs use a variety of V, D, and J germline genes and do not appear to share particular genetic traits required for binding this epitope.^22^, ^71^ Thus, it would be difficult to use an NGS approach to identify bnAbs from new donors even if the serum neutralization was clearly N332 dependent. Interestingly, the initial observation that suggested N332 was a key part of this epitope was that only N332 deletion could completely abrogate neutralization, but it did not always do so for all virus strains and N301 was also implicated.^22^ Further investigation revealed that the high‐mannose patch bnAbs exhibit a degree of promiscuity for different glycan sites across the epitope, allowing them to maintain neutralization breadth in the face of viral changes to glycosylation sites.^28^ The level of permissiveness for different glycan sites varies among the members of this bnAb class, for example, moving the glycan site from 332 to 334 in a six‐virus panel has no effect on PGT128, prevents neutralization of two of four viruses by PGT121, and renders PGT135 unable to neutralize any virus.^28^ Furthermore, within bnAb families, viral variability is tolerated to different degrees due to structural differences. PGT128 and PGT130 belong to branches of the same bnAb family, but due to a six‐residue insertion, PGT128 is better able to accommodate glycan location and heterogeneity in the V1 loop than PGT130.^73^

The third class of bnAbs target the CD4‐binding site (Figure 2) and have been predominantly isolated by a binding‐based selection using proteins designed to isolate bnAbs from donors where this specificity is apparent in the serum neutralization profile.^39^ This approach negates the need to screen thousands of individual B‐cell culture supernatants and allows a more streamlined process for isolating bnAbs. The first CD4‐binding site bnAb isolated, apart from b12,^12^ was VRC01, which partially mimics the binding of CD4 to its receptor site.^74^ The RSC3 bait used to capture the VRC01 B‐cell lineage was modified to preferentially bind b12 and a negative bait that could not bind b12 was used for counter selection.^39^ This strategy was re‐utilized to isolate PGV04 from a separate donor. This bnAb, in contrast to VRC01, does not induce conformational changes in gp120 upon binding.^25^ Many additional CD4‐binding site bnAbs were isolated using RSC3^56^ or other Env baits^40^ and one by EBV immortalization of B cells followed by an ELISA‐based binding screen.^75^ Structural studies have enabled comparison of the CD4‐binding site bnAbs and the definition of two subclasses: those that bind predominantly using their CDRH3 and those that are genetically restricted and use either the VH1‐2 or VH1‐46 V gene.^76^ Within the genetically restricted subclass, the potent VRC01‐like antibodies use only the VH1‐2 V gene and also share an unusual short light chain motif, unlike VH1‐46 V gene bnAbs.^57^, ^76^, ^77^ Recently, it has been shown that while VRC01‐like heavy chains can mature relatively rapidly, generation of light chains that are able to accommodate glycans obstructing access to the epitope takes longer.^78^ In contrast, the CD4‐binding site bnAbs that bind via their CDRH3 are drawn from a wide variety of V genes, have no conserved binding motif, but approach the trimer from similar angles.^76^ In summary, the CD4 binding site is recognized by bnAbs that show, in detail, divergent modes of binding but that are clustered around the two molecular solutions described above.^76^

The fourth class of bnAb is a highly divergent set, derived from multiple donors by a variety of methods, but all members of the class target the gp120‐gp41 interface (Figure 2). Despite this, the epitopes are not completely overlapping and many do not compete with one another in the same way that apex and high‐mannose patch bnAbs do, which is in agreement with negative‐stain microscopy data showing their distribution across the trimer interface.^47^ Many gp120‐gp41 interface bnAbs have been isolated in rapid succession over the last few years,^24^, ^37^, ^47^, ^48^ and some previously identified bnAbs^44^ have been shown to bind to this region.^45^, ^79^ The first bnAb shown to bind this region was PGT151 and was again isolated by our large‐scale screen of single B‐cell culture supernatants for neutralization activity.^24^ This bnAb is highly specific for cleaved pre‐fusion Env and potently neutralizes via interaction with complex tri‐ and tetra‐antennary glycans at positions 611 and 637 within gp41^24^ and protein residues K490, T499, R500, R503 in gp120.^80^ Shortly after, bnAb 35022 was isolated, also by selecting for neutralization activity, and found to be trimer‐specific. It also binds the gp120‐gp41 interface although at a site closer to the viral membrane than PGT151.^37^ 35022 is predicted to be orientated parallel to the membrane and, unlike PGT151, is not cleavage specific.^37^ Coincidental with the discovery of these two new specificities, two previously identified bnAbs, 3BC315 and 3BC176, for which the epitope was not originally delineated^44^ were found to bind to an area partially overlapping with 35022.^45^ However, unlike 35022, the binding of these two bnAbs results in destabilization of the trimer. Similarly, a bnAb isolated using a gp120 bait strategy, 8ANC195^40^ was found to also bind the gp120‐gp41 interface, and to bind to glycans at 276, 234, and 637, with a footprint falling between those of PGT151 and 35022.^37^, ^43^ Strikingly, this bnAb can bind to Env both in a closed conformation and partially reverse the open‐conformation induced by concomitant CD4 binding.^79^ Most recently, two additional trimer‐specific bnAbs, ACS202 and N123‐VRC34.01, were isolated and found to bind to yet another distinct part of the gp120‐gp41 interface and to contact the fusion peptide.^47^, ^48^ The differences between the members of this class of bnAbs, particularly with regard to different conformational requirements for trimer binding, highlight how a bait strategy based on any one of these observations alone may have reduced the likelihood of isolating the other gp120‐gp41 interface bnAbs.

The fifth class of bnAbs comprise those which target the MPER (Figure 2); namely 10E8, which was selected by single B‐cell culture and screening for neutralization^36^; and 2F5 and 4E10, which were isolated by a hybridoma approach.^16^, ^18^, ^19^, ^81^ The latter two Abs were isolated prior to PG9/16 and although they have quite extensive breadth are not as potent as most of the Abs described herein as bnAbs. Interestingly, 4E10, and to a much lesser degree 2F5, exhibit polyreactivity in vitro^82^, ^83^, ^84^ but were shown to be protective and non‐pathogenic during an in vivo challenge study.^85^ However, 2F5 and 4E10 transgenic mice have greatly impaired B‐cell development as 95% of cells fail to complete the pre‐B to immature B‐cell transition.^86^ The minority of B cells which circumvent this checkpoint are anergic, but yet can still be activated by an MPER immunogen.^86^ The more recently isolated MPER bnAb 10E8 is highly potent and does not display any autoreactivity nor does it bind lipids as has been reported for other MPER Abs.^87^ This may be because 10E8 approaches MPER from an altered angle and uses a different binding mode to 4E10.^36^ Serum analysis suggested 10E8‐like specificities were not unusual, with 27% of 78 donors exhibiting this activity. However, to date, only one potent MPER bnAb has been identified, and additional work is needed to isolate more MPER bnAbs so that this area of vulnerability can be more fully understood by defining differences between MPER bnAbs as is underway for the other bnAb classes.

## 4 Conclusions

Given the great progress over the past decade in isolating bnAbs and studying their modes of action, an obvious question is do we still need more bnAbs? The more recent discovery and characterization of gp120‐gp41 interface bnAbs suggest it is still worthwhile to search for new bnAbs because they could reveal novel sites of vulnerability on Env. Furthermore, even if new bnAbs are only subtly different to those currently identified, defining these differences can substantially improve our mechanistic understanding of each bnAb class. This in turn will help us to understand how to induce such bnAbs by immunization and how to evaluate if any similar responses or precursors are stimulated by current immunogens.

The advances in Ab isolation methods over the last decade have made a huge contribution to the discovery of such a large number of HIV bnAbs in a relatively short‐time period. Therefore, another important question is what is the best way to improve this technology in order to seek out new bnAbs and possibly novel epitopes? There are considerable advantages, in terms of cost and time, to reducing the number of B cells that are screened. Thus far, this has been achieved by pre‐enriching for Env‐specific B cells by single‐cell FACS. The development of native‐like stabilized trimers has greatly improved our ability to use this method to select for B cells that bind the functional Env trimers as compared with non‐functional forms of Env. However, even with stabilized near‐native trimers as FACS baits, non‐neutralizing binders and strain‐specific nAbs are captured.^88^, ^89^ Also, it is important to note that inherently a binding screen will select the best binders for the particular assay used. In the case of single‐cell FACS, the assay involves multimeric Abs (as the B‐cell receptors on the surface of B cells) binding to a streptavidin tetramer bound to biotinylated gp120 or stabilized trimer. This is a somewhat different situation to free dimeric soluble antibody binding to low‐density functional Env spikes on virion as occurs during viral neutralization. Therefore, improvements in bnAb isolation methods would be useful in order to combine the streamlined approach of pre‐enriching for Env‐specific B cells with a more informative screen for neutralization function, without the need to culture tens of thousands of single B cells.

The further development of Ab isolation methods is not only important to help identify novel bnAbs but also to evaluate Abs induced by candidate immunogens. Until recently, very few immunization studies had induced even Tier‐2 autologous neutralization, let alone a broad response.^90^, ^91^, ^92^, ^93^ Post‐immune neutralization titers are lower than those seen in elite neutralizer bnAb donors, thus neutralizing Abs may be less frequent among the post‐immunization B‐cell population. Thus far, bnAb‐like Abs have not been seen post‐Env immunization^88^, ^90^, ^94^ except in transgenic mouse models^95^, ^96^, ^97^, ^98^, ^99^ and camelids.^100^, ^101^ However, isolation of neutralizing Abs from immunized rabbits has already provided an explanation for the limited serum neutralization breadth observed to date with one near‐native Env preparation^88^, ^93^: namely, that isolate‐specific glycan holes can be highly immunogenic and the target of the majority of the autologous Tier 2 serum responses observed.^88^ Therefore, it seems probable that, without a major improvement in the potency and breadth of the serum response, establishing whether even a small proportion of the response is bnAb‐like will require higher resolution Ab isolation methods with even greater throughput.

Following the identification of PG9 and PG16 in 2009 and the many other bnAbs that came after, it has become possible to design immunogens based on our knowledge of these extraordinary antibodies. The hope is that by tailoring immunogens to elicit bnAbs rather than strain‐specific and non‐neutralizing Abs, it will be possible to elicit broad and potent immune responses.^3^, ^102^ The growing library of bnAbs provides valuable information for vaccine design efforts, complemented by emerging data on the kinds of neutralizing Abs induced by germline targeting molecules and near‐native stabilized trimers in genetically outbred animals and transgenic mice. In conclusion, despite the progress made to date in identifying bnAbs, new approaches are now needed for two interlinked reasons. First, to increase throughput in order to determine if extremely rare HIV bnAbs precursors are induced by immunization. Second, to identify more bnAbs against known specificities and discover novel epitopes that otherwise might be overlooked when evaluating post‐immunization responses.
